# Skin Friction: Mechanical and Tribological Characterization of Different Papers Used in Everyday Life

**DOI:** 10.3390/ma16165724

**Published:** 2023-08-21

**Authors:** Luís Vilhena, Luís Afonso, Amílcar Ramalho

**Affiliations:** Centre for Mechanical Engineering, Materials and Processes (CEMMPRE), Department of Mechanical Engineering, University of Coimbra, 3030-788 Coimbra, Portugal

**Keywords:** human skin, coefficient of friction, paper, mechanical properties, relative humidity

## Abstract

The coefficient of friction for different contacting materials against skin is mainly influenced by the nature of the materials (synthetic and natural fabrics), mechanical contact parameters (interfacial pressure and sliding velocities), and physiological skin conditions (ambient humidity and skin moisture content). In the present research work, seven different types of papers used in everyday life were analyzed. The physical properties of these materials were determined through tensile tests and friction tests. By comparing mechanical properties with coefficient of friction, it was possible to conclude that the coefficient of friction is strongly correlated with the mechanical properties.

## 1. Introduction 

Studies on mechanical properties of the human skin, performed by several authors [1,2,3,4,5,6,7,8,9], revealed that the human skin behaves as a heterogeneous, anisotropic, and nonlinear viscoelastic material, mainly due to its layered structure (epidermis, dermis, and subcutaneous tissue), where the deformation processes that occur in the skin exhibit a time-dependent behavior. 

With regard to the influence of location and orientation on the mechanical properties of the skin (anisotropy), several research groups performed studies to investigate these factors [10,11,12], and it was found that mechanical properties of skin are dependent upon the orientation of Langer lines and the orientation of collagen fibers in the dermis.

Knowledge of the tribological behavior of human skin in contact with other materials is of great importance as it allows the optimization of material surfaces in contact with the skin. Although the skin has a complex structure, in fact, when it is analyzed in relation to its tribological behavior, a simplification is made. It is considered that the surface of the skin is in contact with the surface of a material in the presence of a possible “lubricant” and with certain external conditions [13]. This simplification means that it is not required to know the structure of materials in detail to obtain some optimization of their frictional behavior. Through this type of parametric approach, some variables are changed to verify their behavior and acquire knowledge in biotribology.

Considering the Amontons-Coulomb law, which states that the frictional force is directly proportional to the normal force and independent of the contact area, Johnson et al. [8] stated that is not valid for the entire range of forces applied to the skin. Comaish S. et al. [9] also established that the behavior of skin deviates from the Amontons-Coulomb law with a more complex relationship of the type F = _μ_W^n^. This is probably because the skin is subject to viscoelastic rather than purely plastic deformation. To describe the tribological behavior of human skin, the theoretical concepts applied to elastomers should be considered [14]. These concepts imply a two-phase friction model, adhesion (total force required to break the adhesive bonds between the two surfaces in contact) and deformation (force related to the deformation of bodies in contact). According to Adams et al. [15], adhesion is considered the main contributor to friction on human skin, rather than the deformation mechanisms that are less relevant. Mattei et al. [16] demonstrated that the lipid layer present on the skin surface would be responsible for the adhesion effect.

Considering the influence of different materials on friction, there are some publications on friction studies carried out between human skin and different types of materials. Derler and Gerhardt [17] and Van Der Heide et al. [13] carried out a literature review and gathered several results of dynamic friction coefficients for the different materials tested against the skin surface. Polymers, metals, glass, fabrics, and paper, among others, were analyzed. The values of the friction coefficients presented varied between 0.09 and 2.7. Regarding the test conditions, the maximum normal force exerted was approximately 4 N for the forearm and 25 N for the index finger, where the anatomical areas analyzed were the ventral surface of the forearm and the index finger. Other body regions were also evaluated. The palm of the hand has also been studied, although it has not been the subject of as much investigation as the ventral side of the forearm and the index finger. Through a first analysis of the results presented, it was verified that there is an obstacle in terms of comparisons between the results obtained. Since each study was performed under different experimental conditions, it becomes difficult and ambiguous to compare the coefficient of friction between two or more materials. However, for tests carried out under similar conditions or within the same study, one can get an idea about which materials have a higher friction value and vice versa. Since biotribology is a relatively recent area, and there are not many studies on this topic yet, the use of standards is practically non-existent. As far as the authors are aware, there are some studies about the physical properties of paper, and in some of these studies, tests of tactile perception are also performed [18,19,20,21]. Although Gee et al. [20] and Skedung et al. [19] had both performed friction tests on printing paper, they obtained different values for the friction coefficient. It is necessary to consider that the papers were possibly not the same; however, the difference in results is essentially due to the different experimental conditions and procedures. Apparently, the main factor that may have influenced the results in a more emphasized way was the applied normal load. While in the study by Gee et al. [20] the normal load ranged from 2 to 20 N, in the study by Skedung et al. [19] a load between 0.2 and 5.2 N was used. In both investigations, friction was measured with the fingertip. The applied loads in these studies may not have been chosen in the best way, because, according to Ramalho et al. [22], for higher loads, fingertip friction presents a behavior characterized by two regimes. Furthermore, according to Skedung et al. [19], this transition between regimes (at the tip of the finger) occurs at approximately 2 N. Similar behavior has also been verified in the ventral face of the forearm, whereas in the palm of the hand, friction presents only one regime [23]. These differences in behavior may be related to the thickness of the skin.

In relation to operating conditions, studies performed by several research groups [24,25,26,27,28,29] found that the friction of skin strongly depends on the contact conditions, like moisture. Other studies investigated gender, age, and anatomical sites [8,24,25,26,27,28,29]. In general, each individual showed a highly positive linear correlation between skin moisture and the coefficient of friction. Related to gender, the friction of female skin showed significantly higher moisture sensitivity when skin hydration varied between very dry and normally moist skin.

More recently, our research team [30,31] investigated the friction of human skin on different fabrics for medical use. The results showed that the friction coefficient of a reference hospital tissue on the skin is influenced by the region of the human body and by the lubrication conditions and physiology of the skin, such as the moisture content. For the different anatomical regions, the coefficient of friction in wet skin exceeded the coefficient of friction in natural skin conditions by a factor of more than two, with friction increasing with increasing moisture content. The use of Vaseline also increased the friction coefficient when compared to the skin’s natural conditions.

The aim of the present research work is to investigate the mechanical and tribological properties of different papers used in everyday life situations and the construction of a comfort map, comparing the physical measured properties and the tactile properties evaluated by a group of volunteers.

## 2. Experimental 

### 2.1. Specimens

**Figure 1:**
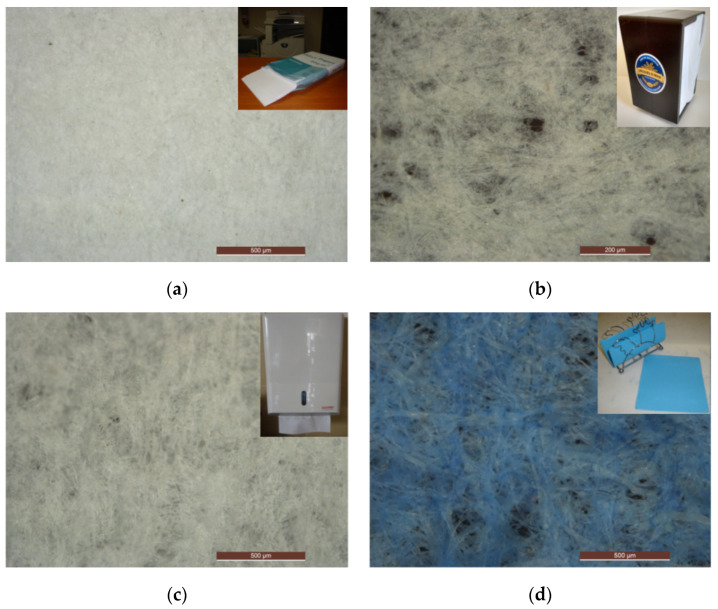

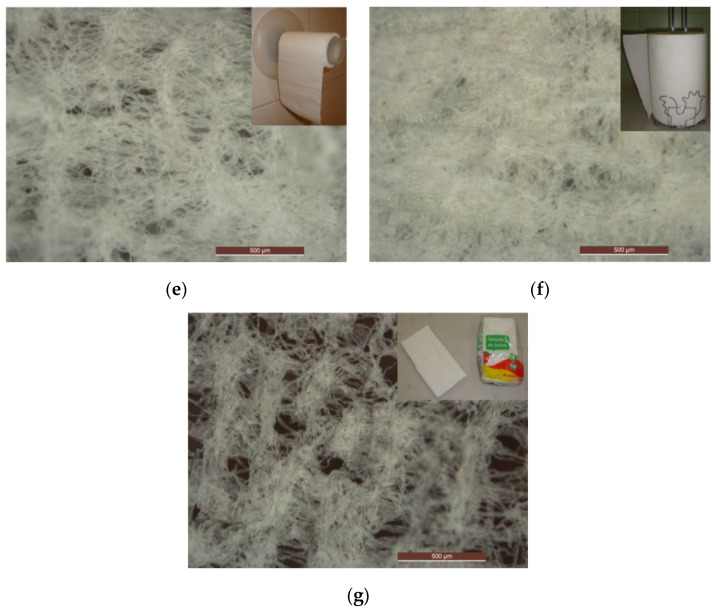
Micrographs of different papers for personal use: (**a**) printing paper used as a reference, (**b**) bar type napkin, (**c**) hand towels paper, (**d**) napkin paper, (**e**) toilet paper, (**f**) kitchen towels paper, (**g**) handkerchiefs paper.

To carry out the experimental work, seven different paper specimens were selected. One of the specimens corresponds to printing paper, and the remaining six specimens correspond to different types of paper used for personal hygiene. The printing paper was selected for the present study because it has been used in several research studies [19,20] and can be used as a reference. The different specimens were selected in an attempt to cover a wide range of the most varied type of papers used in daily personal hygiene, for which it is possible to qualify tactile perception and quantify physical properties. In Figure 1, the seven selected specimens are presented. It is also possible to observe a micrograph image of each sample, obtained through an optical microscope (LED OM Leica DM 4000 M, Leica, Wetzlar, Germany). Table 1 shows the grammage or “weight of paper”, thickness, and density that were calculated in our lab. The thickness was determined with two glass slides and a caliper.

### 2.2. Mechanical and Tribological Characterization Tests

**Figure 2:**
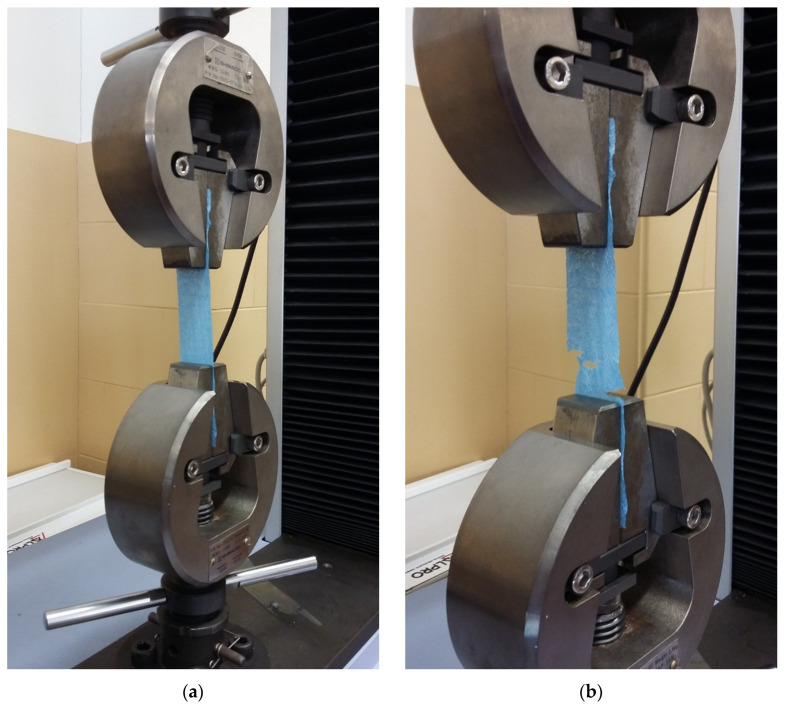
Tensile test at Shimadzu universal testing machine: (**a**) before the paper brakes; (**b**) after paper breakage.

The machine used to carry out the tensile tests is a Shimadzu Autograph AG-X-5kN universal testing machine (Shimadzu, Kyoto, Japan) (Figure 2) with a 5 kN load cell. This equipment was operated at a constant strain rate. Regarding the preparation of the samples, the samples were rectangular in section and had the following dimensions: 25.4 ± 0.5 mm in width, 50 ± 0.5 mm in length between grips, and 120 to 150 ± 0.5 mm in total length (e.g., for toilet paper it was not possible to obtain samples with a total length of 150 mm because of the limitation of the length of each paper strip. However, this variation in the total length had no influence on the results, as the most important thing is the length between grips, which was kept constant throughout the study). Prior to the sketch for later cutting of the samples, the conditions in which each type of paper was found were verified in relation to anomalies such as creases, holes, wrinkles, or other characteristics not typical of the paper itself that could negatively affect the results obtained. The tests were carried out in the longitudinal direction, at a deformation rate corresponding to 7.1 mm/min ± 0.1%. Subsequently, tests were carried out at a deformation rate of 25.4 mm/min ± 0.1% in order to compare the effect of changing the deformation rate on the mechanical properties. Five tests were carried out for each type of paper, that is, all the average parameters obtained by carrying out the tensile tests correspond to an average of five repetitions.

The tensile tests were performed according to the ASTM D828 [32] which describes the procedure for determining four tensile-breaking properties of paper: tensile strength, tensile energy absorption, tensile stiffness, and elongation at rupture. Tensile tests were performed at room temperature using standard tensile wedge grips, and no slippage was observed since the stress-strain curves showed a predictable behavior. Experimental details can be accessed elsewhere [32,33]. One factor (type of paper) ANOVA analysis was performed on the mechanical properties for 7.1 mm/min and is presented in Appendix A.

The friction between the skin and the seven different types of papers was measured in vivo in two distinct anatomical regions (the palm of the hand and the ventral surface of the forearm) and all tests were performed by only one individual (male, Caucasian, and 24 years old). All tests were performed in the longitudinal direction and with the skin under normal conditions, that is, prior to the tests, there was no addition of moisturizing creams or other products to the skin that could alter its hydration level. 

**Figure 3:**
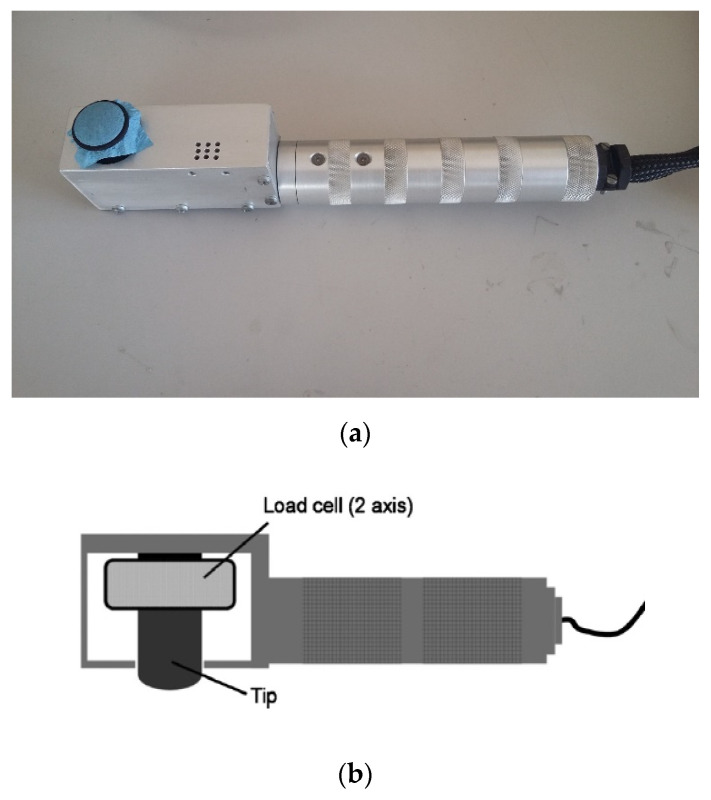
Portable measuring probe to measure skin friction: (**a**) a picture showing the O-ring and a type of paper with blue color and, (**b**) a schematic picture of the multi-component force sensor.

As can be seen in Figure 3, the tests were performed by sliding the paper samples, fixed to the tribometer through an O-ring, over the skin surface in the anatomical region under study. This equipment is manually operated and therefore some care was needed to always keep the probe normal to the skin surface and perpendicular to the sliding direction in order to minimize errors. The surface of the PVC probe on which the paper sample is placed has a smooth spherical surface to avoid edge effects. In addition to the probe, this tribometer is based on a two-axis force sensor as shown in Figure 3b. The force sensor is composed of two load cells that are based on the variation of the Ohmic resistance of a strain gauge. The normal force is measured on one axis and the tangential force on the other, which were exerted on the probe during the tests. The tribometer is connected to an analog-to-digital, A/D, conversion board, so that data can be acquired by the computer during the tests. 

The sliding velocity was constant and equal to 60 ± 10 mm/s, whereas the normal load applied was increasing, with its maximum taking the value of 11 ± 2 N. While in the palm of the hand, a sliding distance of 75 ± 5 mm was used; on the ventral surface of the forearm, this distance was 105 ± 5 mm. The only parameter that varied during the tests was the relative humidity of the paper. The influence of this parameter on the dynamic friction coefficient was studied. For this, tests were carried out at three different relative humidities on all types of papers. Initially, the tests were carried out at an ambient relative humidity of 55 ± 10%, and later on, tests were carried out at relative humidity of 32 ± 2% and 83 ± 2%. A different salt was used for each desired relative humidity. Potassium acetate (CH_3_COOK) was used to obtain a relative humidity of 32 ± 2%, while potassium sulfate (K_2_SO_4_) was used to obtain a relative humidity of 83 ± 2%. These salts were placed in containers, and deionized water was added to form a paste. Three tests were carried out for each type of paper and relative humidity, thst is, all the average parameters obtained by carrying out the tribological tests correspond to an average of three repetitions.

To verify whether certain properties would be correlated with each other, Spearman correlation analyses were performed. Spearman’s *ρ* coefficient assesses the intensity of the relationship between two ordinal variables. Instead of the analyzed parameter values, this coefficient uses only their order. This correlation coefficient varies between –1 and 1, and the closer it is to these extremes, the stronger the correlation of the parameters analyzed. For values close to 1, it means that there is a strong positive correlation, that is, the variables vary in the same direction. For values close to −1, it means that there is a strong negative correlation, that is, the variables vary in opposite directions. To determine the Spearman coefficient *ρ*, the following expression is used (Equation (1)):

(1) $$\rho =1-\frac{6\sum d^{2}}{n_{2}^{2}-1}$$

where *n*_2_ corresponds to the total number of data pairs used in the statistical analysis, and *d* represents the sum of differences in ordinal values associated with the analyzed parameters.

## 3. Results and Discussion

### 3.1. Tensile Tests 

**Figure 4:**
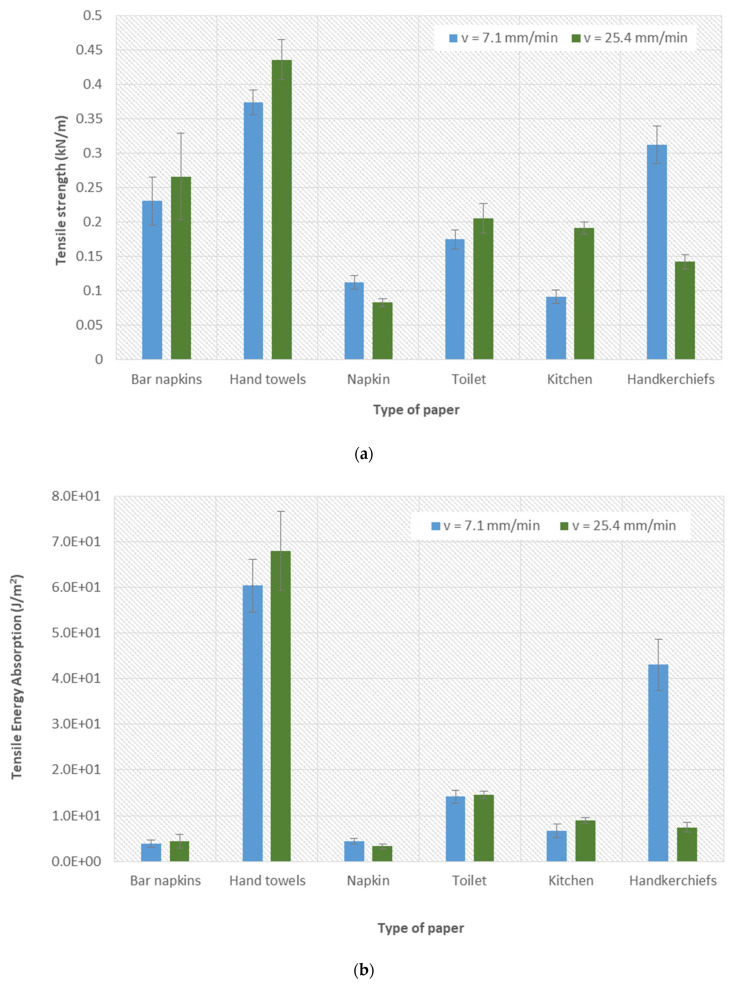

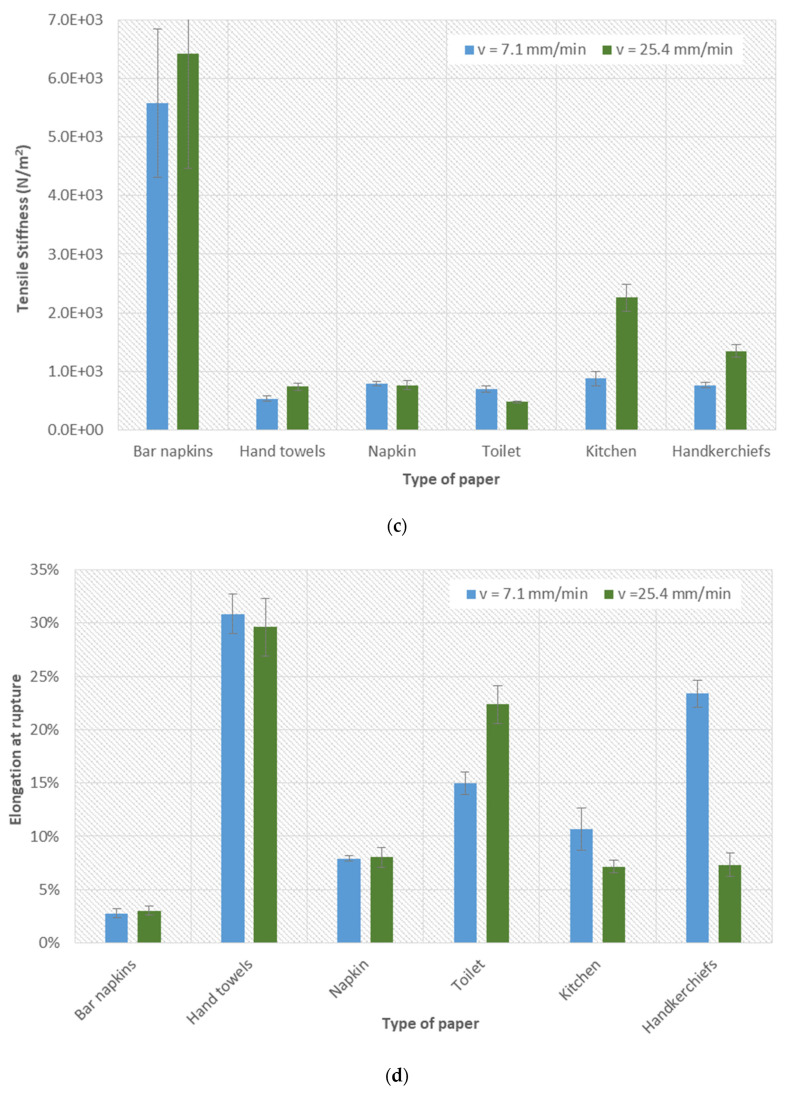
Tensile tests for the six different papers under two different rates of elongation (7.1 and 25.4 mm/min): (**a**) tensile strength; (**b**) tensile energy absorption–TEA; (**c**) tensile stiffness; and (**d**) elongation at rupture.

Tensile tests were performed at two different elongation speeds: 7.1 and 25.4 mm/min. Therefore, it was possible to make a comparison between these two sets of tests, and in this way, the influence of speed on the determined mechanical properties was evaluated. In the figures presented below, Figure 4a illustrates the variation of the tensile strength for different types of papers at two different velocities. Through the analysis of the results, it was possible to conclude that the tensile strength was, on average, 27% higher for a speed of 25.4 mm/min, compared to a speed of 7.1 mm/min. The higher the test speed, the greater the resistance that a material offers to tension. Figure 4d shows the elongation at rupture obtained for the different types of papers at two different velocities.

Through the analysis of the results, it was possible to conclude that the elongation at rupture was, on average, 15% higher for a speed of 7.1 mm/min, compared to a speed of 25.4 mm/min. At lower speeds, different paper samples tend to distort more. Regarding the energy absorption by traction, Figure 4b illustrates its result for the different operating speeds according to each type of paper. For the speed of 25.4 mm/min, the value of tensile energy absorption was, on average, 1.4% higher, compared to the speed of 7.1 mm/min. Finally, the tensile stiffness was also analyzed in relation to the effect of the speed of the tensile tests (Figure 4c). Through the results obtained, it was possible to conclude that, for a speed of 25.4 mm/min, the tensile stiffness presents values 59% higher compared to the values obtained for a speed of 7.1 mm/min. As with tensile strength, the higher the test speed, the more rigid a material is under tension.

Since the printing paper, used as a reference, presented values for the mechanical properties at least one order of magnitude higher than all the other papers, it was decided to indicate these values in Table 2.

### 3.2. Tribological Tests

**Figure 5:**
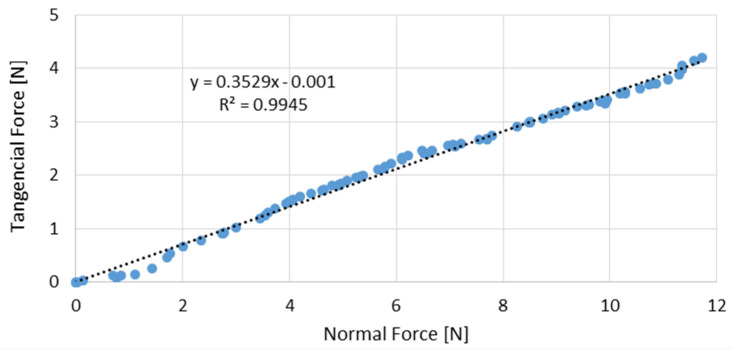
Evolution of the tangential force with the normal force for the handkerchiefs rubbing against the volar forearm for RH = 55% (blue dots are the experimental points while the dotted line represent the linear correlation equation).

Friction tests were performed for each different type of paper at three different relative humidities (32, 55, and 83%) and at two different anatomical regions, the palm of the hand and the ventral forearm. Figure 5 shows an example where the coefficient of friction was determined for the handkerchiefs rubbing against the volar forearm for RH = 55%. The value of the coefficient of friction corresponds to the slope of the trend line that represents the evolution of the tangential force as a function of the normal force (μ = 0.35). The slope was obtained using the simple linear regression model and considering the Amontons-Coulomb friction model.

**Figure 6:**
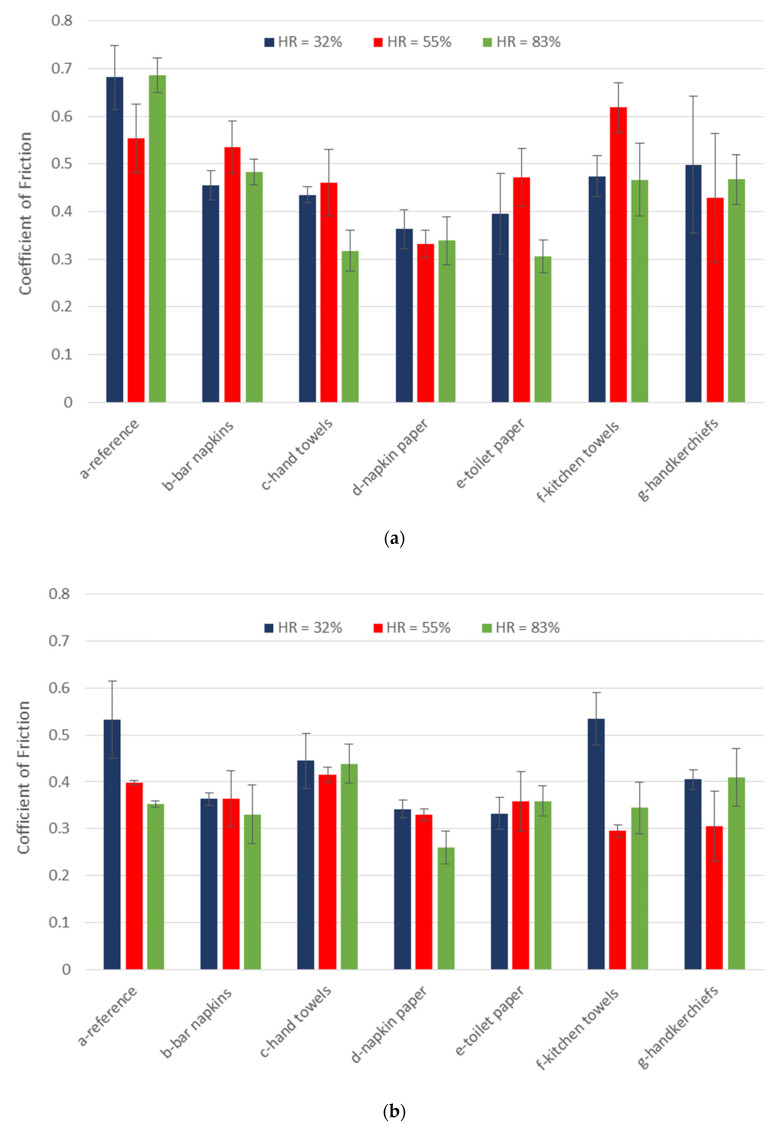
Coefficient of friction for the seven different papers at three different relative humidities (RH = 32, 50, and 83%) measured at: (**a**) the palm of the hand; (**b**) the ventral forearm.

Figure 6 shows the coefficient of friction for the seven different papers rubbing against the palm of the hand (Figure 6a) and the ventral forearm (Figure 6b) for three different relative humidities. It can be seen that the COF varies between a minimum of 0.26 and a maximum of 0.68. Through the analysis of Figure 6, it also appears that in fact, the coefficient of friction is higher in the palm area when compared to the ventral surface of the forearm. This behavior occurs in all types of paper, and on average, the coefficient of friction in the palm of the hand is 25% higher than the coefficient of friction in the forearm. These results are in agreement with Ramalho et al. [34]. A possible justification is that the palm of the hand is an area with higher roughness and/or higher moisture content than the forearm due to the amount of sweat excreted in this area being higher. Also, the properties of the skin, mainly the thickness and stiffness, can explain the results obtained [34]. In the tests in which the paper was tested with a relative humidity of 32% and 83%, this type of behavior was also maintained, however, to a lesser extent.

It is observed that, in general, the friction of human skin increases with the level of moisture [30,31,34,35]. However, by observing Figure 6, no obvious trend can be seen about the behavior of skin friction rubbing against different papers for different relative humidities. This must be related to the thickness and moisture absorption capacity of the different papers.

### 3.3. Correlation between Coefficient of Friction and Mechanical Properties

**Figure 7:**
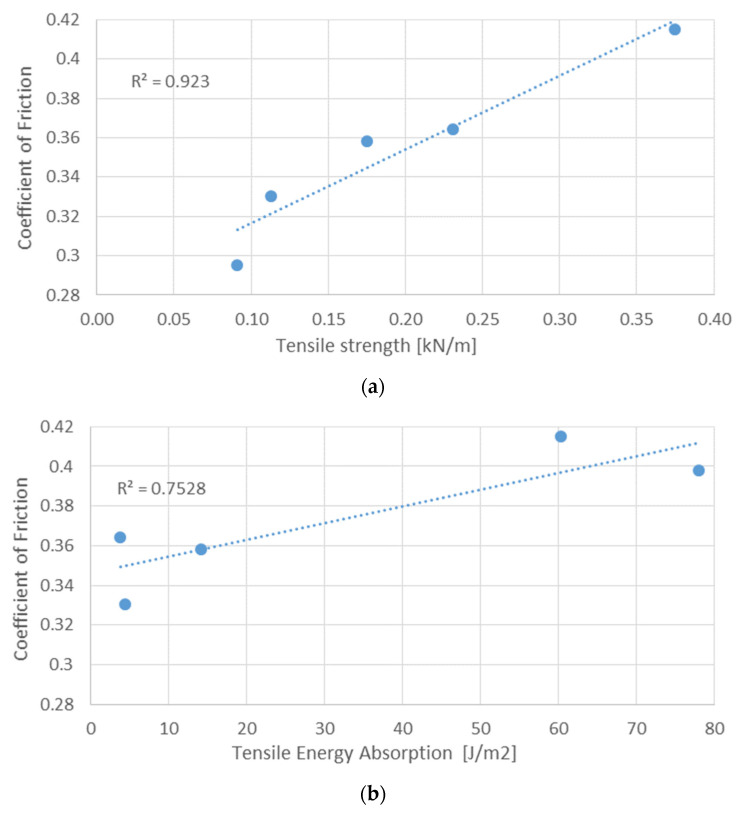

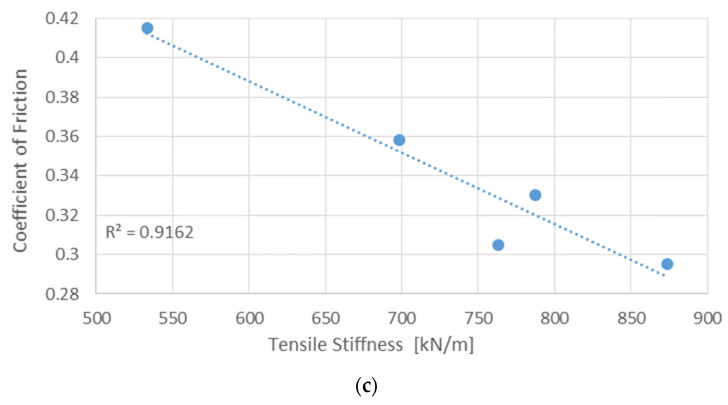
Correlation between the coefficient of friction and different mechanical properties: (**a**) tensile strength; (**b**) tensile energy absorption – TEA; and (**c**) tensile stiffness (blue dots are the experimental points while the dotted line represent the linear correlation equation).

The mechanical properties obtained through the tensile tests were compared with the coefficient of friction, namely the tensile strength, the tensile energy absorption, and the tensile stiffness. The coefficient of friction was measured at the volar forearm for RH = 55%. No significant correlations were obtained between the coefficient of friction and the various mechanical properties. However, for the tensile strength, a strongly positive Spearman coefficient of 0.94 was obtained, excluding samples (a) and (g), of printing paper and handkerchief paper, respectively. This may indicate that an increase in tensile strength may result in an increase in the coefficient of friction (Figure 7a). Concerning the tensile energy absorption, a positive Spearman coefficient of 0.60 was obtained, excluding samples (f) and (g), kitchen towels, and handkerchief paper, respectively. This correlation is no longer as strong as the previous one. Nevertheless, there is a certain tendency that seems to indicate that the increase in energy absorption by traction leads to an increase in the coefficient of friction (Figure 7b). For the tensile stiffness, a strongly negative Spearman coefficient of −0.90 was obtained, excluding samples (a) and (b) of printing paper and bar-type napkins, respectively. This means that with increasing tensile stiffness, the coefficient of friction decreases (Figure 7c). 

## 4. Conclusions

The coefficient of friction between the skin sliding against seven different types of papers for personal use was evaluated. For this, a portable friction measuring probe was used. The coefficient of friction was determined in two anatomical regions, the palm of the hand and the ventral surface of the forearm. Each paper was tested at three different relative humidities: 32, 55, and 83%. Tensile tests were also carried out in which it was possible to determine the following mechanical properties: tensile strength, strain at break, tensile stiffness, and tensile energy absorption:

- It has been shown that the coefficient of friction is higher in the palm area when compared to the ventral surface of the forearm. On average, the coefficient of friction in the palm of the hand was 25% higher compared to the ventral surface of the forearm;
- The effect of the relative humidity on the coefficient of friction was not evident. This fact must be related to the thickness and moisture absorption capacity of the different tested papers.

Regarding the relation between the mechanical properties and coefficient of friction:

- A strongly positive Spearman coefficient of 0.94 was obtained between the coefficient of friction and the tensile strength. The increase in tensile strength leads to an increase in the coefficient of friction;
- A strongly negative Spearman coefficient of −0.90 was obtained between the coefficient of friction and tensile stiffness. This means that with the increase in tensile rigidity. The coefficient of friction tends to decrease.

## Figures and Tables

**Table 1:** Grammage (“weight of paper”), thickness, and density of the different papers used during experiments.

Type of Paper	Designation	Grammage (g/m^2^)	Thickness (mm)	Density (g/cm^3^)
Printing paper (sheet)	a	84.22	0.23	0.37
Bar type napkins (dispenser)	b	18.64	0.06	0.31
Hand towels paper (dispenser)	c	34.44	0.16	0.22
Napkin paper	d	20.37	0.16	0.12
Toilet paper	e	32.31	0.27	0.12
kitchen towels paper	f	42.82	0.65	0.07
Handkerchiefs paper	g	62.63	0.23	0.27

**Table 2:** Mechanical properties obtained in the tensile tests for the printing paper (used as a reference).

Elongation Speed (mm/min)	Tensile Strength (kN/m)	Tensile Energy Absorption (J/m^2^)	Tensile Stiffness (N/m^2^)	Elongation at Rupture (%)
7.1	1.95 ± 0.08	78.01 ± 11.35	60.56 ± 4.08	5.5 ± 0.6
25.4	4.52 ± 3.02	122.67 ± 27.78	151.49 ± 95.56	4.9 ± 1.7

## Data Availability

The data presented in this study are available on request from the corresponding author. The data are not publicly available due to privacy.

## Appendix A

**Table A1:** Tensile Energy Absorption-ANOVA: One factor and seven groups (Factor: Type of paper).

Anova: Single Factor						
SUMMARY						
*Groups*	*Count*	*Sum*	*Average*	*Variance*		
Printing	5	0.277204	0.055441	3.72 × 10^−5^		
Bar napkins	5	0.137902	0.02758	1.7 × 10^−5^		
Hand towels	5	1.537188	0.307438	0.000268		
Napkin	5	0.395608	0.079122	5.65 × 10^−6^		
Toilet	5	0.748145	0.149629	0.000117		
Kitchen	5	0.531785	0.106357	0.000392		
Handkerchiefs	5	1.168416	0.233683	0.000165		
ANOVA						
*Source of Variation*	*SS*	*df*	*MS*	*F*	*p-value*	*F crit*
Between Groups	0.307348	6	0.051225	357.6672	4.05 × 10^−25^	2.445259
Within Groups	0.00401	28	0.000143			
Total	0.311358	34				

**Table A2:** Elongation at rupture-ANOVA: One factor and seven groups (Factor: Type of paper).

Anova: Single Factor						
SUMMARY						
*Groups*	*Count*	*Sum*	*Average*	*Variance*		
Printing	5	390.0481	78.00962	128.7637		
Bar napkins	5	19.16648	3.833295	0.590042		
Hand towels	5	301.9262	60.38524	33.56738		
Napkin	5	22.20864	4.441729	0.377227		
Toilet	5	70.82795	14.16559	2.028468		
Kitchen	5	33.49638	6.699277	2.254712		
Handkerchiefs	5	215.288	43.05759	31.36285		
ANOVA						
*Source of Variation*	*SS*	*df*	*MS*	*F*	*p-value*	*F crit*
Between Groups	27,651.06	6	4608.509	162.1537	2.05 × 10^−20^	2.445259
Within Groups	795.7776	28	28.42063			
Total	28,446.83	34				

**Table A3:** Tensile strength-ANOVA: One factor and seven groups (Factor: Type of paper).

Anova: Single Factor						
SUMMARY						
*Groups*	*Count*	*Sum*	*Average*	*Variance*		
Printing	5	9.761677	1.952335	0.007036		
Bar napkins	5	1.154109	0.230822	0.001223		
Hand towels	5	1.872931	0.374586	0.000328		
Napkin	5	0.564382	0.112876	9.85 × 10^−5^		
Toilet	5	0.874139	0.174828	0.000189		
Kitchen	5	0.455905	0.091181	9.76 × 10^−5^		
Handkerchiefs	5	1.561798	0.31236	0.000746		
ANOVA						
*Source of Variation*	*SS*	*df*	*MS*	*F*	*p-value*	*F crit*
Between Groups	13.23203	6	2.205338	1588.397	4.09 × 10^−34^	2.445259
Within Groups	0.038875	28	0.001388			
Total	13.2709	34				

**Table A4:** Tensile Stiffness-ANOVA: One factor and seven groups (Factor: Type of paper).

Anova: Single Factor						
SUMMARY						
*Groups*	*Count*	*Sum*	*Average*	*Variance*		
Printing	5	302,802.2	60,560.43	16,617,676		
Bar napkins	5	27,874.71	5574.942	1,611,219		
Hand towels	5	2665.55	533.1099	1685.899		
Napkin	5	3935.249	787.0499	1585.967		
Toilet	5	3491.031	698.2062	2664.365		
Kitchen	5	4368.631	873.7263	15,403.88		
Handkerchiefs	5	3814.473	762.8946	1875.543		
ANOVA						
*Source of Variation*	*SS*	*df*	*MS*	*F*	*p-value*	*F crit*
Between Groups	1.5 × 10^10^	6	2.5 × 10^9^	960.5731	4.53 × 10^−31^	2.445259
Within Groups	73,008,442	28	2,607,444			
Total	1.51 × 10^10^	34				

General Conclusion: Since the *p*-value is approximately zero, we reject the hypothesis of equality of means for any significance level. There are significant differences between the various mechanical properties of the various types of analyzed paper.

## References

[1] Dupuytren G. (1834). Traite Theorique et Pratique des Blessures par Armes le Guerre.

[2] Langer A.K. (1861). Zuranatomie und Physiologie der haut. Sber. Akad. Wiss. Wien.

[3] Langer K. (1861). On the Anatomy and Physiology of the Skin.

[4] Fung Y.C.B. (1964). Elasticity of soft tissues in single elongation. Am. J. Physiol..

[5] Lanir Y., Fung Y. (1974). Two-dimensional mechanical properties of rabbit skin—II. Experimental results. J. Biomech..

[6] Alexander H., Cook T. (1977). Accounting for natural tension in the mechanical testing of human skin. J. Investig. Dermatol..

[7] Tong P., Fung Y.-C. (1976). The stress-strain relationship for the skin. J. Biomech..

[8] Johnson S., Gorman D., Adams M., Briscoe B. (1993). The friction and lubrication of human stratum corneum. Tribol. Ser..

[9] Comaish S., Bottoms E. (1971). The skin and friction: Deviations from Amontons’ laws, and the effects of hydration and lubrication. Br. J. Dermatol..

[10] Annaidh A.N., Bruyère K., Destrade M., Gilchrist M.D., Otténio M. (2012). Characterising the Anisotropic Mechanical Properties of Excised Human Skin. J. Mech. Behav. Biomed. Mater..

[11] Delalleau A., Josse G., Lagarde J.M., Zahouani H., Bergheau J.M. (2008). A nonlinear elastic behavior to identify the mechanical parameters of human skin in vivo. Skin Res. Technol..

[12] Wilkes G., Brown I., Wildnauer R. (1973). The biomechanical properties of skin. CRC Crit. Rev. Bioeng..

[13] Van Der Heide E., Zeng X., Masen M.A. (2013). Skin tribology: Science friction?. Friction.

[14] Dowson D. (1997). Tribology and the skin surface. Bioengineering of the Skin: Skin Surface Imaging and Analysis.

[15] Adams M.J., Briscoe B.J., Johnson S.A. (2007). Friction and lubrication of human skin. Tribol. Lett..

[16] Pailler-Mattei C., Nicoli S., Pirot F., Vargiolu R., Zahouani H. (2009). A new approach to describe the skin surface physical properties in vivo. Colloids Surf. B Biointerfaces.

[17] Derler S., Gerhardt L.-C. (2011). Tribology of Skin: Review and Analysis of Experimental Results for the Friction Coefficient of Human Skin. Tribol. Lett..

[18] Thieulin C., Pailler-Mattei C., Vargiolu R., Lancelot S., Zahouani H. (2017). Study of the tactile perception of bathroom tissues: Comparison between the sensory evaluation by a handfeel panel and a tribo-acoustic artificial finger. Colloids Surf. B Biointerfaces.

[19] Skedung L., Danerlöv K., Olofsson U., Johannesson C.M., Aikala M., Kettle J., Arvidsson M., Berglund B., Rutland M.W. (2011). Tactile perception: Finger friction, surface roughness and perceived coarseness. Tribol. Int..

[20] Gee M., Tomlins P., Calver A., Darling R., Rides M. (2005). A new friction measurement system for the frictional component of touch. Wear.

[21] Skedung L., Danerlöv K., Olofsson U., Aikala M., Niemi K., Kettle J., Rutland M.W. (2009). Finger Friction Measurements on Coated and Uncoated Printing Papers. Tribol. Lett..

[22] Ramalho A., Silva C.L., Pais A.A.C.C., Sousa J.J.S. (2006). In vivo Friction Study of Human Palmoplantar Skin against Glass. Tribologia.

[23] Ramalho A., Silva C., Pais A., Sousa J. (2007). In vivo friction study of human skin: Influence of moisturizers on different anatomical sites. Wear.

[24] Gerhardt L.L.-C., Strässle V., Lenz A., Spencer N., Derler S. (2008). Influence of epidermal hydration on the friction of human skin against textiles. J. R. Soc. Interface.

[25] Hendriks C.P., Franklin S.E. (2009). Influence of Surface Roughness, Material and Climate Conditions on the Friction of Human Skin. Tribol. Lett..

[26] Nacht S., Close J.-A., Yeung D., Gans E.H. (1981). Skin friction coefficient: Changes induced by skin hydration and emollient application and correlation with perceived skin feel. J. Soc. Cosmet. Chem..

[27] Denda M., LodeA M., Maibach N.H. (2000). Dry Skin and Moisturizers: Chemistry and Function.

[28] Wolfram L.J. (1983). Friction of skin. J. Soc. Cosmet. Chem..

[29] Hills R.J., Unsworth A., Ive F.A. (1994). A comparative study of the frictional properties of emollient bath additives using porcine skin. Br. J. Dermatol..

[30] Vilhena L., Ramalho A. (2016). Friction of Human Skin against Different Fabrics for Medical Use. Lubricants.

[31] Vilhena L.M., Ramalho A. (2019). Friction Behavior of Human Skin Rubbing against Different Textured Polymeric Materials Obtained by a 3D Printing Microfabrication Technique. Tribol. Trans..

[32] (2022). Standard Test Method for Tensile Properties of Paper and Paperboard Using Constant-Rate-of-Elongation Apparatus.

[33] Fernandes E.F.d.M. (2018). Estudo das Condições de Contacto Com Atrito Envolvendo a Pele Humana.

[34] Ramalho A., Ludema K.C., Shaffer S.J. (2009). Influence of Environmental Humidity on the Friction of Human Skin Against Textiles. Proceedings of the 17th International Conference on Wear of Materials.

[35] Tomlinson S.E., Lewis R., Liu X., Texier C., Carre M. (2010). Understanding the Friction Mechanisms Between the Human Finger and Flat Contacting Surfaces in Moist Conditions. Tribol. Lett..

